# Distribution, Source and Risk Assessment of Heavy Metal(oid)s in Water, Sediments, and Corbicula Fluminea of Xijiang River, China

**DOI:** 10.3390/ijerph16101823

**Published:** 2019-05-23

**Authors:** Xuexia Huang, Dinggui Luo, Dongye Zhao, Ning Li, Tangfu Xiao, Jingyong Liu, Lezhang Wei, Yu Liu, Lirong Liu, Guowei Liu

**Affiliations:** 1 School of Environmental Science and Engineering, Guangzhou University, Guangzhou 510006, China; huangxuexia66@163.com (X.H.); tfxiao@gzhu.edu.cn (T.X.); wlz2016@gzhu.edu.cn (L.W.); liuyu@gzhu.edu.cn (Y.L.); leonliutiming@e.gzhu.edu.cn (L.L.); liuguowei@e.gzhu.edu.cn (G.L.); 2 Linköping University—Guangzhou University Research Center on Urban Sustainable Development, Guangzhou University, Guangzhou 510006, China; 3 Guangdong Provincial Key Laboratory of Radionuclides Pollution Control and Resources, Guangzhou University, Guangzhou 510006, China; 4 Key Laboratory for Water Quality and Conservation of the Pearl River Delta, Ministry of Education, Guangzhou University, Guangzhou 510006, China; 5 Environmental Engineering Program, Department of Civil Engineering, Auburn University, Auburn, AL 36849, USA; zhaodon@auburn.edu; 6 Guangxi Zhuang Autonomous Region Environmental Monitoring Station, Nanning 530028, China; lining1972@sohu.com; 7 Ecological Environment Information System and Big Data Research Team, Guangdong University of Technology, Guangzhou 510006, China; liujycust@163.com

**Keywords:** metal, water pollution, sediment contamination, *corbicula fluminea*, risk assessment, metalloid

## Abstract

A total of 43 water and sediment samples, and 34 *Corbicula fluminea* samples were collected in Xijiang River in southern China to determine the spatial distribution and sources of 12 metals/metalloids (V, Co, Cr, Ni, Cu, Mn, Zn, Cd, Pb, As, Sb, and Tl) and to assess the pollution levels and ecological risks of the pollutants. The results showed that the levels of the metals/metalloids (except for Tl) in the river water from almost all of the sampling sites met the Chinese national surface water quality standards. However, the concentrations of the metals/metalloids in the sediments exceeded the background values by a factor of 1.03–56.56 except for V, Co, and Mn, and the contents of Zn, Cd, and Pb in the *Corbicula fluminea* soft tissue exceeded the limits of the Chinese Category I food Quality Standards. The spatial distribution analysis showed that the concentrations of the contaminants in the lower reaches of Xijiang River were higher than in the upper reaches. The bioaccumulation factor (*BAF*), biota-sediment accumulation factor (*BSF*), geo-accumulation index (*I_geo_*), and the potential ecological risk index (*RI*) were obtained to assess the pollution levels and ecological risks. The results indicated that Cu, Cd, and Zn were the most prone to bio-accumulation in the *Corbicula fluminea* soft tissue, and the lower reaches showed a much higher pollution level and risk than the upper reaches. The metals/metalloids in the sediments posed serious threat on the aquatic ecosystem, of which Cd, As, and Sb are the most risky contaminants. The results of principal component analysis (PCA) indicated Cr, Ni, Cu, Mn, Cd, Pb, and As in the sediments came from relevant industrial activities, and V and Co originated from natural sources, and Sb from mining activities, Zn and Tl came from industrial activities and mining activities.

## 1. Introduction

With the rapid development of economy and the continuous expansion of city size, the contamination of water, biotas, and sediments by metals and metalloids has become a serious environmental issue in many countries [1,2,3,4]. The released metals/metalloids can pose serious and long-lasting threats to the environment due to the associated adverse health effect, environmental persistence, and bioaccumulation in biotas and human body. While trace amounts of metals/metalloids can originate from natural sources, such as weathering of rocks and minerals and volcanic activities, human activities account for the most input into the aquatic systems, including mining and mineral processing [5,6], metal smelting [7,8], domestic and industrial effluents [9,10], urban surface runoff [11,12], agriculture and animal husbandry activities [13,14], fossil fuel combustion and atmospheric depositions [15,16], and ship dumping [17].

Typically, the majority of released metals/metalloids accumulate in the surficial sediment in aquatic systems via adsorption, chelation, and sedimentation processes [18], and about 10% of them exist in the water column in the dissolved form [19,20], or are associated with suspended particles. Yet, heavy metals/metalloids in the sediments may be released into the water column by physical, chemical, and biological remobilization processes [21]. As such, sediments are usually considered to be the major sink of heavy metals/metalloids in aquatic ecosystems. Furthermore, the sediments also play very important roles in the aquatic safety and in assessing the potential ecological risk of the pollutants in the aquatic environment.

*Corbicula fluminea* (*C. fluminea*) is a freshwater bivalve mollusk and it is widely distributed in freshwater lakes and rivers worldwide [22]. It has been used as a bio-indicator in the monitoring of contamination and bioavailability of heavy metals in aquatic ecosystems over the past 30 years [23]. Its ubiquitous distribution and sedentary habit ease the sampling. Moreover, *C. fluminea* lives in the water-sediment interface and it may act as a sedimentary filter feeder, with the ability to accumulate heavy metals that are dissolved in water or bound to fine sediments [24], which facilitates the chemical analysis [25].

Xijiang River is the major tributary of the Pearl River, ranking as the second largest river in China in terms of average streamflow next to the Yangtze River. It covers 78% of the Pearl River drainage basin and provides 64% of the water discharge. Xijiang River flows through Wuzhou, Zhaoqing, Yunfu, Foshan, Jiangmen, Zhongshan, Zhuhai City, and it finally enters into the South China Sea. It is a major source of drinking water for 86 counties with a total population of 28.7 million and it also supplies water for industries and agricultural purposes in the Guangxi and Guangdong Provinces [26]. Located in a region of rapid economic growth, Xijiang River has suffered from serious environmental degradation in recent decades, owing to the massive mining and industrial activities. Large amounts of wastewater containing heavy metals/metalloids without effective treatment were discharged from the mines, urban areas, and sewage systems into the river. It was reported that the discharge of mining wastes had caused serious Tl and Cd pollution of Xijiang River [27]. High concentrations of heavy metals, including Zn, Cd, Cr, Cu, and Pb, have been detected in the river surface sediments of upper reaches of the river basin [28]. However, to date, most studies regarding heavy metals in the Pearl River basin have been focused on Pearl River or Beijiang River, which is another major tributary of the Pearl River. Little information is available regarding the contents of heavy metals in the water and sediments of Xijiang River, especially on the relationship of heavy metals concentration in water, sediments, and *C. fluminea*.

Thus, the main objectives of this study were to: (1) determine total contents of V, Co, Cr, Ni, Cu, Mn, Zn, Cd, Pb, As, Sb, and Tl in the water, sediments, and *C. fluminea* of Xijiang River; (2) evaluate the major sources of the heavy metals/metalloids and, (3) assess the potential ecological risks that are posed by these heavy metals/metalloids.

## 2. Materials and Methods

### 2.1. Description of the Sampling Area and Sample Collection

The Xijiang River basin is located in a region of subtropical-tropical monsoon climate (102°14′–113°33′ E, 21°31′–26°49′ N) in southern China. The annual mean temperature is 14–22 °C and the annual average precipitation is 1000–2000 mm, and about 80% of rain falls occur in the wet season (from April to September). The mainstream of Xijiang River originates from the Maxiong Mountains of Yunnan Province, and it flows through Guizhou, Guangxi, and Guangdong provinces from northwest to southeast. The study area belongs to the lower reaches of the mainstream, it covered a length of 350 km of Xijiang River, and the average annual runoff is 2.33 × 10^11^ m^3^. The non-ferrous metal mineral resources in the Xijiang River basin are very abundant, especially the reserves of Pb, Zn, Tl, and Sb rank first in China.

**Figure 1:**
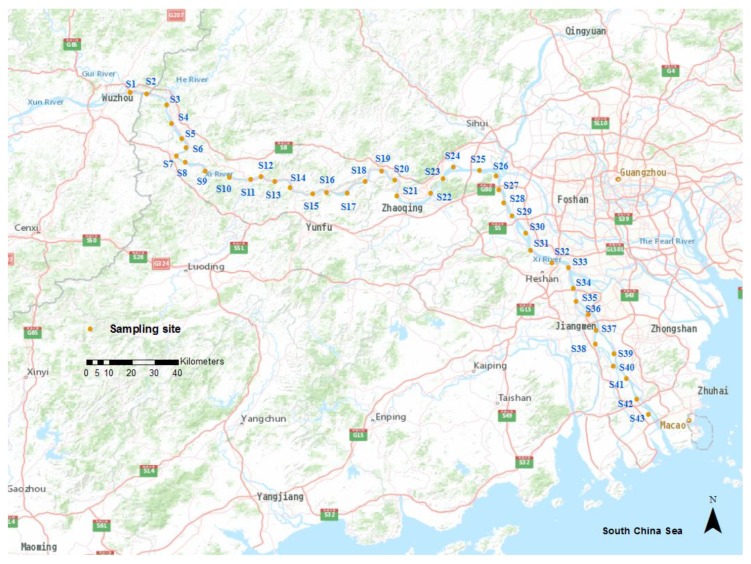
Map of the sampling sites.

Water, sediments, and *C. fluminea* samples were collected from 43 sites along Xijiang River on July 9 2015 (Figure 1 and Table S1 in the Supplementary Materials). At each sampling site, three subsamples of water or sediment were collected and combined as one representative sample. The water samples were collected with 100 mL syringes from about 0.15 m below the water surface and sealed in 500 mL polyethylene plastic bottles. The pH values of water samples were measured in the field with a portable pH meter. Meanwhile, sediment samples (top 10 cm) at each corresponding site were collected while using a stainless-steel grab sampler and kept in sealed polyethylene bags. *C. fluminea* samples (shell length between 20 and 25 mm) were separated from the collected sediments, with exception of the sampling sites S1, S7, S11, S15, S20, S30, S33, S37, and S41, and held in high-density polyethylene containers that were filled with the site water. As such, a total of 43 water, sediment, and 34 *C. fluminea* samples were collected from Xijiang River. All of the samples were kept in ice boxes and transported to the laboratory for further treatment and analysis.

### 2.2. Sample Preparation and Analysis

The water samples were filtered through 0.45 μm disposable filter membranes (Whatman GmbH, Dassel, Germany) and acidified (pH < 2) using analytical grade nitric acid and then stored in coolers at 4 °C for metal analysis.

The sediment samples were freeze-dried and sieved through a 2-mm nylon sieve to remove any stones and coarse debris. The sieved samples were ground and passed through a 0.149-mm nylon sieve. Subsequently, 0.1 g of each sediment sample was digested with 8 mL of 68% nitric acid (HNO_3_, *v*/*v*) and 3 mL of 40% hydrofluoric acid (HF, *v*/*v*). 

For the *C. fluminea* samples, the soft tissues were taken out of the shells and then rinsed with Milli-Q water. The soft tissue was freeze-dried and measured to obtain the dry body weight. Afterwards, 0.5 g of the homogenized freeze-dried soft tissue was acid-digested with a mixture of HNO_3_ (68%, *v*/*v*) and hydrogen peroxide (H_2_O_2_, 30%, *v*/*v*). 

The digestion process was conducted with an automatic digestion system (Polytech ST40, Polytech Instrument Ltd., Beijing, China). The digestant was then filtered through a 0.45 μm membrane filter. Consequently, the concentrations of V, Co, Cr, Ni, Cu, Mn, Zn, Cd, Pb, As, Sb, and Tl in the water and the digestant samples were analyzed using a Perkin-Elmer ELAN6100 inductively coupled plasma mass spectrometry (ICP-MS) (PerkinElmer Inc., Waltham, MA, USA).

### 2.3. Quality Assurance and Quality Control

Strict quality control measures were applied to all the analyses, including the uses of triplicate samples, reagent blanks, and certified reference materials, to control the quality of chemical analyses and assure the accuracy of the experimental data. The certified reference materials of GBW07446 (for Chinese soils) and GBW10024 (for scallops) were used to confirm the analytic methods. The recoveries of heavy metals extracted via the GBW-07446 and GBW-10024 procedures ranged from 93.4% to 107.5% and from 95.7% to 107.1%, respectively, with a relative standard deviation (RSD) of less than 4.5% and 5.8%. All of the polyethylene and glass bottles were soaked in HNO_3_ solution for 24 h and then thoroughly rinsed with deionized water before use. 

### 2.4. Statistical Analysis

The Pearson correlation coefficients were calculated to determine the correlations among the heavy metal concentrations in the water, sediment, and biota samples. The criterion for significance in the correlation analysis was set at the level of *p* < 0.01 (significant). The method of principal components analysis (PCA) with varimax rotation of standardized component loadings was used to identify the possible distribution and sources of the heavy metals/metalloids. The number of principal components was determined based on the Kaiser criterion with an eigenvalue that is greater than 1 [29]. All of the statistical analyses were conducted with the SPSS 19 and the figures were produced using OriginPro 9.1 for Windows (OriginLab Corporation, Northampton, NC, USA). The geographic information system software ArcGIS 10.3 (Environmental Systems Research Institute Inc., Redlands, CA, USA) was used to draw the map of the sampling sites.

### 2.5. Risk Assessment

The bioaccumulation factor (*BAF*) [30], biota-sediment accumulation factor (*BSF*) [31], geo-accumulation index (*I_geo_*) [32], the potential ecological risk factor for a single metal/metalloid (*E_r_^i^*), and the potential ecological risk index (*RI*) [33] were calculated based on the field data to assess the potential ecological risk associated with the heavy metals/metalloids. 

*BAF* and *BSF* were calculated by the following equations:

(1) $$BAF=\frac{E_{i}}{D_{i}}$$

(2) $$BSF=\frac{E_{i}}{C_{i}}$$

where, *E_i_*, *D_i_*, and *C_i_* is the average concentrations of metal i in *C. fluminea* (mg kg^−1^), water (mg L^−1^), and sediment (mg kg^−1^), respectively. The values of *BAF* and *BSF* represent the relative ability of *C. fluminea* to bio-accumulate selected heavy metals from water and sediments. If *BAF* is much greater than 100, the aquatic organism has a strong potential to accumulate the metals/metalloids [34].

*I_geo_* has been widely used to assess the heavy metals pollution levels in sediment and soil through comparing the differences between pre-industrial and present concentrations. *I_geo_* was defined according to the following equation:

(3) $$I_{\mathrm{geo}}=\log_{2}\left(\frac{C_{n}}{1.5\times B_{n}}\right)$$

where, *C_n_* (mg kg^−1^) is the average concentrations of metal *n* in sediment and *B_n_* is the geochemical background of metal *n* in China’s continental crust (V = 99, Co = 32, Cr = 63, Ni = 57, Cu = 38, Mn = 780, Zn = 86, Cd = 0.055, Pb = 15, As = 1.9, Sb = 0.15, Tl = 0.61 mg kg^−1^). *I_geo_* is categorized into the following seven classes: extremely polluted (*I_geo_* > 5), strongly to extremely polluted (4 ≤ *I_geo_* ≤ 5), strongly polluted (3 ≤ *I_geo_* < 4), moderately to strongly polluted (2 ≤ *I_geo_* < 3), moderately polluted (1 ≤ *I_geo_* < 2), unpolluted to moderately polluted (0 < *I_geo_* < 1), and unpolluted (*I_geo_* ≤ 0).

*E_r_^i^* and *RI* were calculated to estimate the potential ecological risk of a single metal and mixtures of metals/metalloids in sediment via:

(4) $$E_{r}^{i}=T_{r}^{i}\times \frac{C^{i}}{C_{n}^{i}}$$

(5) $$RI=\sum_{i=1}^{n}E_{r}^{i}$$

where, $T_{r}^{i}$ is the toxic factor of a metal/metalloid (V = 2, Co = 5, Cr = 2, Ni = 5, Cu = 5, Mn = 1, Zn = 1, Cd = 30, Pb = 5, As = 10, Sb = 7, Tl = 10) [35,36,37,38,39]. C^i^ is the measured concentration of metal *i* in sediment samples and *C_n_^i^* is the background value of heavy metal *i*. The background values of heavy metals in soils of China were used in this study [40]. *E_r_^i^* is the corresponding background value of the metal. *E_r_^i^* is categorized into the following five classes: very high risk (*E_r_^i^* > 320), high risk (160 ≤ *E_r_^i^* ≤ 320), considerable risk (80 ≤ *E_r_^i^* < 160), moderate risk (40 ≤ *E_r_^i^* < 80), and low risk (*E_r_^i^* < 40). *RI* is the sum of *E_r_^i^* and it is categorized into four classes: very high ecological risk (*RI* > 600), considerable ecological risk (300≤ *RI* ≤ 600), moderate ecological risk (150 ≤ *RI* < 300), and low ecological risk (*RI* < 150).

## 3. Results and Discussion

### 3.1. Levels of Metals/Metalloids in the River Water 

The pH values of the river water from the 43 sampling sites varied from 6.3 to 7.9 with a mean of 7.2 ± 0.41, which met the national surface water environmental quality standards [41]. Table 1 presents the means, minimums, maximums, and standard deviations of the target metals/metalloids that are determined in the water, sediments, and *C. fluminea* from Xijiang River.

The mean contents of the pollutants in the river water followed the order of: Mn > Zn > Cu > Cr > Ni > As > Pb > Sb > V > Co > Cd > Tl. It is noteworthy that the values of Cr, As, Sb, and Pb in Xijiang River were lower than those in Beijiang River, which is another major tributary of the Pearl River. [42,43] (Table S2 in Supplementary Materials), while the concentrations of V, Mn, Cd, Ni, and Tl were higher than those in Beijiang River, (e.g., contents of V, Mn, and Tl were approximately two to three times higher) [44]. When compared with other rivers in China, the contents of the metals/metalloids in Xijiang River were much higher than those in Jingjiang River (about one to nine times) [45] and Xiangjiang River (about 1 to 20 times) [46], except for V and As. Yet, the contents of Cu, As, Ni, Cr, V, Cd, Pb, and Sb in part of Yangtze River [47] were about three to 100 times higher than those in Xijiang River, except for Zn and Mn, which were about two to four times lower than those in Xijiang River. The relatively higher levels of the metals/metalloids in Xijiang River might be ascribed to industrial discharges. According to the Water Resources Bulletin that ws issued by water resources department of Guangdong Province, from 2013 to 2015, the average annual discharge of wastewater into Xijiang River was 3.42 × 10^8^ tons, of which 51% was industrial wastewater.

**Figure 2:**
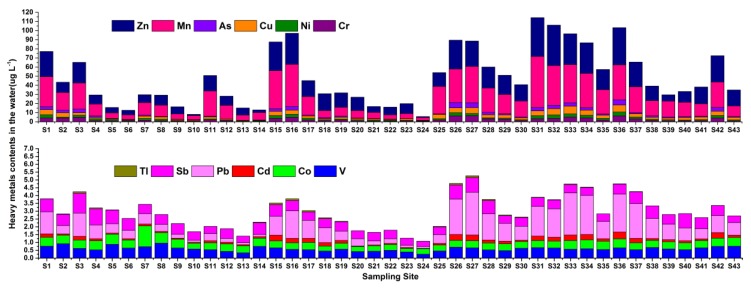
The contents of various metals/metalloids from different sampling sites of Xijiang River.

Figure 2 shows the metals/metalloids contents in the water from different sampling sites. The spatial variation of the metals/metalloids contents along the river exhibited a remarkably increasing trend towards the lower reaches (S26–S43) in contrast with the upper reaches (S1–S25). The higher contents at sampling sites S1, S3, S11, and S15–S17 in the upper reaches were because these sites were located in urban areas (Wuzhou, Feikai, Deqing, and Yunfu City), which have higher population density and industrial discharges, such as metal smelting, electroplating, battery manufacture, printing and dyeing, mining and mineral processing, sulfuric acid manufacture, and wastewater treatment. In addition, high levels of Co, Cr, Mn, Zn, Cd, Pb, As, Sb, and Tl were observed at site S3, which was located at the junction of Xijiang River and Hejiang River. It was reported that a large amount of heavy metals were brought into Xijiang River from Hejiang River [48,49]. In addition, the acid mine drainage from the Yunfu pyrite mining area [50] also discharged a large amount of heavy metals into Xijiang River through Jiangshui River, which caused elevated contents of dissolved Mn, Zn, Cr, Ni, As, Cd, and especially Tl at sites S15–S17.

Sites S26 and S27 were located at the junction of Xijiang River and Beijiang River, and it was known that the latter carried out large amounts of metals/metalloids from a major mining area, the Dabaoshan polymetallic mines [51], into Xijiang [44]. This explains the higher contents of Cr, Pb, Zn, As, Cd, and Tl at these sites. Similarly, elevated contents of Cr, Ni, Cu, Mn, Zn, Cd, As, and Pb were observed at Sites S31–S34, S36, and S42. These sampling sites were located at urban areas (Foshan, Jiangmen, and Zhongshan City), which have been heavily impacted by various major industries involving heavy metals/metalloids, including: circuit board printing (Cu), electroplating and metal surface treating (Cr, Zn, Cu, Ni, Mn, Pb, and Cd), leather tanning and dyeing (Cr and As), building and sanitary ceramic production (Cd, Pb, Cr, Zn, Cu, Ni, and Co), and cadmium-nickel and lead acid battery manufacturing (Cd, Zn, Pb, Cr, Cu, Ni, As, and Mn). Some relevant factories were heavily fined or forced to close for non-compliance or illegal discharges, according to the local Environmental Protection Bureau. It should be noted that the contents of Tl in sampling sites S16 and S27 were slightly higher than the national surface water environmental quality standards [41].

Pearson correlation analysis revealed that the Cu, Mn, Zn, Cr, Ni, Pb, As, and Cd contents in the water were significantly (*p* < 0.01) and positively correlated (0.794 ≤ r ≤ 0.950) with each other (Table S3 in Supplementary Materials), which indicated that these heavy metals/metalloids have the same pollution sources. Overall, anthropogenic activities along Xijiang River have seriously impacted the heavy metal levels in the river water.

### 3.2. Heavy Metals/Metalloids in the Sediments

**Figure 3:**
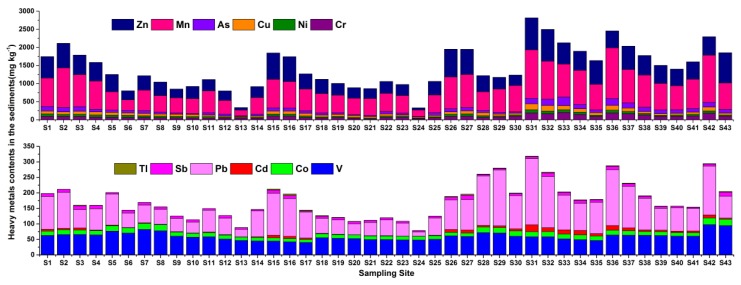
The concentrations of heavy metals/metalloids in the sediments of Xijiang River.

The average contents of the target metals/metalloids in the Xijiang River sediments followed the sequence: Mn > Zn > Cr > Pb > As > Cu > V > Ni > Co > Sb > Cd > Tl (Table 1 and Figure 3). Compared with the background values [40] of those in the sediments of Pearl River (Table S4 in Supplementary Materials), the contents of Ni, Cu, Tl, Pb, As, Sb, and Zn in the Xijiang River sediments were much higher, especially the content of Cd was more than 56 times higher, which indicated that these metals/metalloids pollution might be caused by human activities. The contents of V and Co were slightly lower than those of the background values, which suggested these metals perhaps came from earth crust. In contrast, the mean levels of heavy metals in the Xijiang River were lower than those in the Beijiang River [42,52,53,54] except for Cr, Zn, Ni and Co. This is consistent with a prior study that Beijiang River is the most polluted river by heavy metals among the main tributaries of the Pearl River [43]. However, the mean contents of Cr, Ni, Cu, Pb, Cd, Zn, and As in the Xijiang River sediments exceeded those in the Yangtze River [55], and were much higher than those in the Jinjiang River [45]. Moreover, the mean levels of heavy metals in the Xijiang River sediments were higher than those in the Hejiang River, except for V, Mn, and Sb [56].

As was the case for the river water, the metals/metalloids contents in the sediments of the lower reaches (S26–S43) were 1.1 to 3.9 times higher than in the upper reaches (S1–S25). This is because hundreds of factories involving heavy metals processing were located in the lower reaches (S26–S43), for example, the sampling sites S31 were located at urban areas of Foshan, which have a large number of building ceramic production factories (Zn, Pb, Cu, and Cr) and metal surface treating factories (Mn, Zn, Pb, Ni, and Cd), and these factories have directly or indirectly discharged large quantities of metals-bearing sewage into the river over the past decades. A large fraction of the metals accumulated in the sediments through adsorption, chelation, and sedimentation processes. Although, the local government has taken some measures to reduce the exogenous input of heavy metals, such as controlling the sewage discharge and shutting down some of the main polluters, the heavy metals pollution in the sediments of the Xijiang River is still very serious.

The correlation analysis revealed the contents of Cu, Mn, Zn, Cr, Ni, Pb, As, and Cd in the sediments were significantly (*p* < 0.01) correlated (0.602 ≤ r ≤ 0.904) with each other (Table S5 in Supplementary Materials). Meanwhile, a significant correlation between V and Co (r = 0.824, *p* < 0.01) was determined, which indicated that these two heavy metals may share common sources.

### 3.3. Heavy Metals/Metalloids in C. fluminea

**Figure 4:**
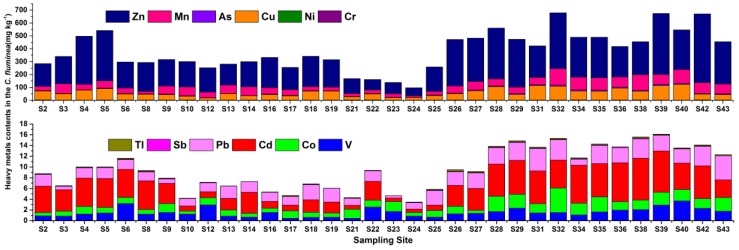
The contents of heavy metals/metalloids in the C. fluminea soft tissue from Xijiang River.

Table 1 and Figure 4 give the levels of the heavy metals/metalloids in the *C. fluminea* soft tissue. The abundance of these metals/metalloids followed the order: Zn > Cu > Mn > As > Cd > Pb > Co > V > Ni > Cr > Tl > Sb. When compared to the Category I food quality standards of aquatic products by the China Food and Drug Administration, the average contents of Cr, As, and Cu in the *C. fluminea* soft tissue were lower than the maximum allowable limits (0.5, 1.0, and 10 mg kg^−1^ w.w., respectively) [57] (note: a conversion factor of 8 was used from wet weight to dry weight [58]). However, the Cu content in the *C. fluminea* soft tissue from 20% of sampling sites exceeded the maximum allowable limit. Furthermore, the average contents of Zn, Cd, and Pb were about one to two times higher than the limits (20, 0.2, and 0.1 mg kg^−1^ w.w., respectively), and Zn, Cd, and Pb at 88.2%, 76.5%, and 94.1% of the sampling sites exceeded the limits. 

The average contents of As, Ni, Cu, Sb, Mn, Co, and Zn in *C. fluminea* from the Xijiang River were higher than that observed in the Odiel River (4.30, 0.99, 44.03, 4.30, 30.77, 0.80, and 128.67 mg kg^−1^ d.w.) of Spain [59], especially the content of Cd was more than five times higher (0.64 mg kg^−1^ d.w.), with the exception of Pb, for which the average concentration was slightly lower (2.98 mg kg^−1^ d.w.). When compared with Altamaha River of USA [60], the soft tissue concentrations of Mn, Cd, Zn, and Pb in *C. fluminea* from the Xijiang River were much higher (51.8, 2.57, 131, and 0.57 mg kg^−1^ d.w.); however, the As, Cu, and Ni were lower than those of the Altamaha River (4.74, 70.0, and 3.88 mg kg^−1^ d.w.).

Like in the river water or sediment, the concentrations of heavy metals/metalloids in the *C. fluminea* soft tissue from the lower reaches (S26–S43) were about 1.14 to 2.48 times higher than those from the upper reaches (S1–S25). This is in accord with the higher concentrations of the metals/metalloids in the water and sediments in the lower reaches (S26–S43), confirming that the contaminant partitioning in *C. fluminea* is associated with the concentrations in the surrounding water and sediments [61]. The content of Mn (1053 mg kg^−1^) in the sediment of sampling sites S32 was not the highest, but the content of Mn (128.7 mg kg^−1^) in *C. fluminea* soft tissue of this sampling site was the highest. This is because the environmental mobility and availability of heavy metals are not only related to the total amount of heavy metals, but, more importantly, they often depend on their chemical forms. Therefore, the contents in the biota may reflect partial bioavailability of the contaminants. Moreover, care needs to be taken, because difference biological species may filter out or resist different metals/metalloids to different extents [62,63]. 

Significant (*p* < 0.01) correlations were found between Mn and Co (r = 0.581), Zn and Cu (r = 0.574), Cd and Cu (r = 0.678), Pb and Zn (r = 0.588), and As and Cd (r = 0.713) in the *C. fluminea* soft tissue (Table S6 in Supplementary Materials), suggesting that these pairs of metals have similar bioaccumulation, common pollution source, and dispersion properties [64].

### 3.4. Correlation Analysis and Source Apportionment

The contents for most of the metals/metalloids in the river water were significantly (*p* < 0.01) correlated (0.566 ≤ r ≤ 0.830) with those in the sediments, regardless of the site locations (Table S7 in Supplementary Materials), which reflects a dynamic local quasi-equilibrium distribution between the two phases [65]. No significant correlations were observed between the metal contents measured in the *C. fluminea* soft tissue and those that were measured in water, which was consistent with a previous study [65]. In contrast, the concentrations of Cu (r = 0.557), Mn (r = 0.484), Zn (r = 0.532), Cd (r = 0.543), Pb (r = 0.615), and As (r = 0.599) in the *C. fluminea* soft tissue showed significant (*p* < 0.01) correlations with the concentrations in the sediments, which again agrees with those by Angelo et al. [65], who observed that the contents of Cd, Pb, and Zn in sediment samples were correlated with those in *C. fluminea* and mussels soft tissues. This is reasonable, because the contact time between *C. fluminea* and the flowing river water is much shorter that that between the biota and the sediments. The partitioning was governed by the sediment phase concentrations as a result.

Physical, chemical, and biological factors, such as organism size, fine sediment fraction, acidity, bioavailability, and the contents of dissolved organic matter, can influence the heavy metals bioaccumulation in *C. fluminea* [60], As such, further study should be conducted to investigate the allocation mechanisms of heavy metals between *C. fluminea* and environmental matrices.

Table 2 shows the results of the principle component analysis (PCA) for the metals/metalloids in the sediments. According to the Kaiser-Meyer-Olkin (KMO) measure of sampling adequacy, a high KMO value (close to 1.0) indicates that a PCA is very useful with a data set, whereas a value of less than 0.50 indicates the PCA may not be suitable. In this work, the calculated KMO value was 0.800 for the sediments data set, and the significance levels of Bartlett’s test of sphericity was 0, which confirms the suitability of the data set for PCA.

**Figure 5:**
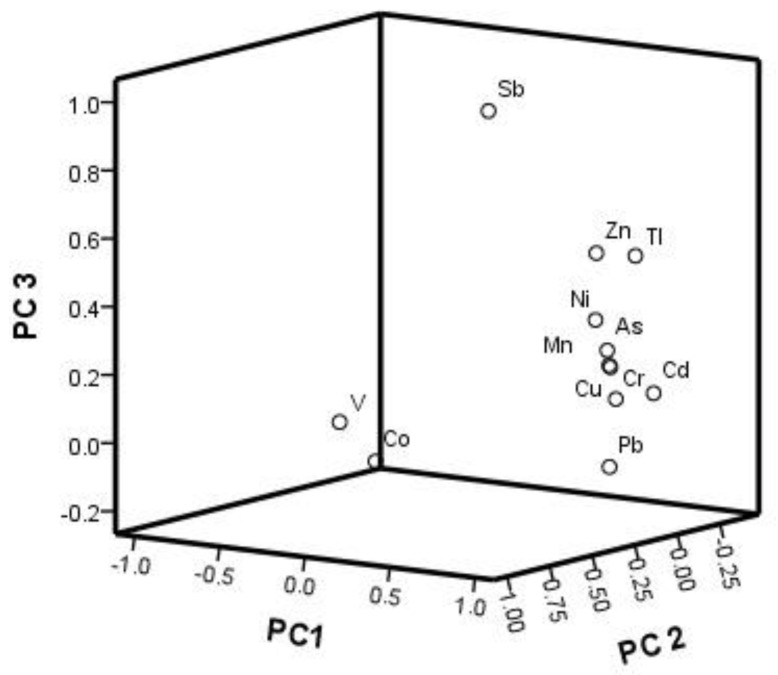
Loading plots of principal component analysis for the three rotated components.

Three principal components of the metals/metalloids in the sediments had eigenvalues greater than 1, with a total variance of 83.310%. The PC1 accounted for 51.98% of the total variance and it had strong positive loadings on Cr, Ni, Cu, Mn, Zn, Cd, Pb, As, and Tl (Figure 5). The PC2 consisting of V and Co represented 16.61% of the total variance, and the PC3 consisting of Zn, Sb, and Tl made up 14.72% of the total variance.

In general, wastewater and sludge from cadmium-nickel and lead acid battery manufacturing plants usually contains Cd, Zn, Pb, Cr, Cu, Ni, As, and Mn [66], whereas Cu is the primary metal for printed circuit boards [67]. Wastewater residue and sludge from electroplating and metal surface treatment industries usually contain Cr, Zn, Cu, Ni, Mn, Pb, and Cd [68], whereas the effluents from leather tanning and dyeing industries usually contain Cr and As [69]. Moreover, the glazes, co-solvents, and colorants that are used in building and sanitary ceramic products often contain Cd, Pb, Cr, Zn, Cu, Ni, and Co [70,71], and the effluents of sulfuric acid plants sometimes contains thallium [72]. 

As all these relevant industries had been actively operating in the study area, the PC1 (Cr, Ni, Cu, Mn, Zn, Cd, Pb, As, and Tl) group seemed to be related to the discharge of industrial wastewater, such as battery manufacturing, electroplating and metal surface treatment, leather tanning and dyeing, ceramic production, and sulfuric acid manufacture. The PC2 (V and Co) seemed to be related to the earth’s crust for their lower contents when compared with the background value. The PC3 (Zn, Sb, and Tl) can be attributed to the Zn, Sb, and Tl mining activities, which is attributed to the active mining in the upper reaches of Xijiang River [56].

### 3.5. Potential Ecological Risk Assessment

Table S8 in Supplementary Materials lists the calculated values of *BAF* and *BSF* for the heavy metals/metalloids in the *C. fluminea* soft tissue. The *BAF* values showed the following sequence: Cd > Cu > Zn > Tl > Co > Mn > As > V > Pb > Sb > Cr > Ni. However, the *BSF* values followed a different sequence: Cu > Cd > Zn > Co > Tl > Mn > As > Pb > Ni > V > Cr > Sb. The *BAF* values of all heavy metals/metalloids were significantly larger than 100, which indicated that these metals/metalloids are bioaccumulative in the *C. fluminea* soft tissue. The *BAF* and *BSF* values of Cu, Cd, and Zn were much higher than those of other metals, suggesting that Cu, Cd, and Zn were much more prone to an accumulation by *C. fluminea*. The result is consistent with the previous studies on bioaccumulation of Cu, Cd, and Zn by *C. fluminea* [73,74]. It is remarkable that the *BAF* value of Tl was relatively high, which was probably because Tl^+^ has a similar radius as K^+^ (1.49 Å for Tl^+^ and 1.33 Å for K^+^) and Tl^+^ can easily substitute for K^+^ in the biochemical processes [75].

**Figure 6:**
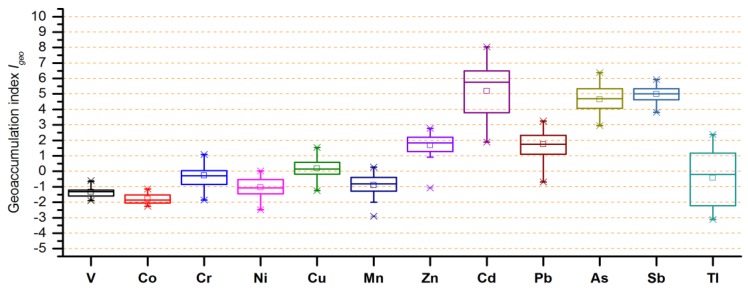
Geo-accumulation index of the studied metals/metalloids in the sediments from Xijiang River.

Figure S1 gives the geo-accumulation index (*I_geo_*) of Cr, Cu, Zn, Cd, Pb, As, Sb, and Tl, and Figure 6 gives the *I_geo_* values for all of the studied metals at all sampling sites. For nearly all of the sites, the *I_geo_* values for V, Co, Ni, and Mn were ≤0, which suggested that the sites were not polluted by these metals. Likewise, Cr was not a pollutant at sampling sites S1–S29, S35, and S39, and only showed slight pollution at other sites. Cu showed moderate pollution at sampling sites S31, S32, and S36, and no pollution or slight pollution at other sampling sites. Tl showed moderate to strong pollution at site S16, and moderate pollution at 11 sampling sites. Notably, Zn and Pb showed moderate to strong pollution at almost all sites of the lower reaches (S26–S43), As and Sb showed strong or extreme pollution in all sampling sites, and Cd showed extreme pollution in all sites of the lower reaches.

**Figure 7:**
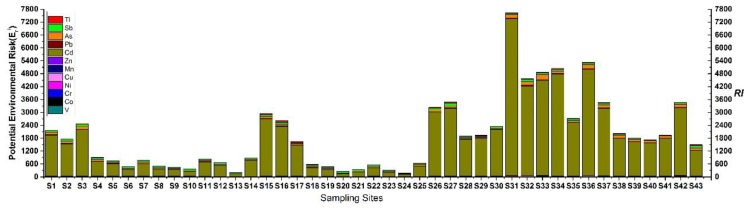
The potential ecological risk (Eri and risk index (RI)) values calculated for various metals/metalloids in the sediments of Xijiang River.

The potential ecological risk was assessed by on the *E_r_* and *RI* values. Figure 7 presents the calculated *E_r_* and *RI* values for the target metals/metalloids in the sediments. Cd, As, and Sb are among the most risky contaminants, where Cd poses high to very high risk at 93% of the sampling sites, As poses moderate to high risk at 83.7% of the sites, and Sb poses moderate to considerable risk at 88.4% of the sites.

Moreover, based on the *RI* values, the lower reaches (S26–S43) and most sites at the upper reaches were under very high ecological risk (*RI* > 600), whereas some of the sites in the upper reaches showed moderate to considerable ecological risk (moderate for S13, S20, and S24; Considerable for S6, S8–S10, S18–S19, and S21–S23).

## 4. Conclusions

Concentrations of V, Co, Cr, Ni, Cu, Mn, Zn, Cd, Pb, As, Sb, and Tl were determined in the water, sediments, and *C. fluminea* from Xijiang River. For the river water, only Tl from two sampling sites exceeded the Chinese national surface water environmental quality standards, despite the heavy industrial impacts on the aquatic system, which suggests the rapid mass transfer of the metals/metalloids from water into the sediment phase and, is, to a certain extent, attributed to the recent government’s efforts in limiting the industrial discharge. However, concentrations of Ni, Cu, Tl, Pb, As, Sb, Zn, and Cd in the sediments were much higher than the background values, which indicated the severe impacts of the industrial activities and the strong sink effect of the sediments. The average contents of Zn, Cd, and Pb in the *C. fluminea* soft tissue were about one to two times higher than the maximum allowable limits of the Category I food quality standards of aquatic products, and Cu, Zn, Cd, and Pb at 20%, 88.2%, 76.5%, and 94.1% of the sampling sites exceeded the limits. The concentrations of Cu, Mn, Zn, Cd, Pb, and As in the *C. fluminea* showed significant correlations with the concentrations in the sediments and significant correlations were found between Mn and Co, Zn and Cu, Cd and Cu, Pb and Zn, and As and Cd in the *C. fluminea*, suggesting that these pairs of metals have a common pollution source (The component score coefficient matrix and scores of principal factors on samples were listed in Tables S9 and S10 in the Supplementary Materials.). Based on the accumulation factor analysis, Cu, Cd, and Zn were the most prone to bioaccumulation by *C. fluminea*. Based on the geo-accumulation index and potential ecological risk index analyses, all of the sites of the lower reaches and 88% sites at the upper reaches of Xijiang River were severely polluted and confronted with very high ecological risk. In particular, Cd, As, Sb, Zn, and Pb in the sediments posed the most serious threat to the aquatic ecosystem.

Based on information from this work, immediate remediation actions should be taken toward these key toxic metals/metalloids in the sediments to mitigate the associated ecological risk.

## Figures and Tables

**Table 1:** Total metal/metalloids contents in water, sediments, and *C. fluminea* of Xijiang River.

Metal	Water (µg L^−1^, *n* = 43)				Sediments (Dry Weight, mg kg^−1^, *n* = 43)				*C. fluminea* (Dry Weight, mg kg^−1^, *n* = 34)			
	Mean	Min	Max	S.D	Mean	Min	Max	S.D	Mean	Min	Max	S.D
V	0.60	0.25	0.96	0.14	59.8	39.9	97.7	12.5	1.54	0.46	3.69	0.80
Co	0.48	0.23	1.33	0.16	14.14	10.12	21.58	2.97	1.56	0.56	4.58	0.83
Cr	2.37	0.29	6.67	1.79	88.43	26.19	200.13	45.23	1.03	0.25	1.97	0.46
Ni	1.73	0.44	4.20	1.05	45.24	15.25	87.37	17.47	1.16	0.24	2.24	0.52
Cu	3.08	0.45	8.42	2.06	70.43	24.22	166.10	30.81	59.5	18.1	121.9	28.6
Mn	20.9	3.1	55.9	13.8	689.5	155.9	1405.0	282.9	58.5	11.8	128.7	28.5
Zn	18.34	1.11	44.31	11.29	466.0	61.0	884.9	195.8	259.4	61.7	533.3	110.7
Cd	0.17	0.02	0.44	0.11	5.09	0.31	21.83	4.77	4.02	0.62	7.79	2.22
Pb	1.03	0.12	2.73	0.75	87.82	14.00	213.66	46.23	2.33	0.45	4.50	0.99
As	1.72	0.11	5.73	1.63	83.30	22.09	237.0	46.17	4.66	1.14	9.87	2.41
Sb	0.65	0.34	1.27	0.18	7.54	3.16	13.73	2.48	0.07	0.00	0.14	0.04
Tl	0.03	0.00	0.11	0.03	1.21	0.11	4.75	1.13	0.11	0.01	0.29	0.07

**Table 2:** Total variance explained, rotated component matrix and principal component loadings for the sediments samples of Xijiang River.

	Initial Eigenvalue			Rotation Sums of Squared Loadings				Principal Component		
	Total	% of Variance	Cumulative%	Total	%of Variance	Cumulative%		PC1	PC2	PC3
1	7.152	59.597	59.597	6.238	51.982	51.982	V	0.070	0.943	0.116
2	1.788	14.898	74.494	1.993	16.605	68.587	Co	0.250	0.912	0.009
3	1.058	8.816	83.310	1.767	14.723	83.310	Cr	0.914	0.165	0.139
4	0.671	5.588	88.897				Ni	0.802	0.172	0.365
5	0.374	3.116	92.014				Cu	0.875	0.158	0.229
6	0.291	2.425	94.439				Mn	0.878	0.169	0.236
7	0.206	1.714	96.153				Zn	0.693	0.058	0.540
8	0.170	1.421	97.573				Cd	0.947	−0.024	0.135
9	0.125	1.044	98.617				Pb	0.881	0.169	−0.062
10	0.074	0.613	99.230				As	0.817	0.119	0.270
11	0.056	0.463	99.693				Sb	0.149	0.148	0.936
12	0.037	0.307	100.000				Tl	0.559	−0.305	0.479

## Supplementary Materials

The following are available online at https://www.mdpi.com/1660-4601/16/10/1823/s1, Figure S1: Box plots of geo-accumulation index of 8 metals/metalloids in the sediments from Xijiang River, Table S1: Longitudinal and latitudinal positions of the sampling sites, Table S2: Metals/metalloids concentrations in water from open publications (μg L^−1^), Table S3: Correlation analysis among different metals/metalloids in the water samples (*n* = 43), Table S4: Metals/metalloids concentrations in sediments from open publications (mg kg^−1^ d. w.), Table S5: Correlation analysis among different metals/metalloids in the sediment samples (*n* = 43), Table S6: Correlation analysis among different metals/metalloids in the Corbicula fluminea samples (*n* = 34), Table S7: Correlations between Corbicula fluminea soft tissue and single metal/metalloid in water and sediment (*n* = 34), Table S8: Geo-accumulation index, potential ecological risk, and average bioconcentration factors (BAF) and biota-sediment accumulation factors (BSF) for metals/metalloids in Corbicula fluminea soft tissue from the Xijiang River, Table S9: Component Score Coefficient Matrix, Table S10: Scores of principal factors on samples.
