# Supplementary material for: Distribution, Source and Risk Assessment of Heavy Metal(oid)s in Water, Sediments, and Corbicula Fluminea of Xijiang River, China
Source: Int J Environ Res Public Health. 2019 May 23;16(10):1823. doi: 10.3390/ijerph16101823 (PMC6572011; doi:10.3390/ijerph16101823)
Supplement: Supplementary file 1 [file ijerph-16-01823-s001.pdf]

International Journal of
Environmental Research and Public Health

MDPI

Supplementary Material

Distribution, Source and Risk Assessment of Heavy Metal(oid)s in Water, Sediments, and Corbicula Fluminea of Xijiang River, China

Xuexia Huang [1,2,3,4], Dinggui Luo [1,2,3,4,*], Dongye Zhao [5], Ning Li [6], Tangfu Xiao [1], Jingyong Liu [7], Lezhang Wei [1,2], Yu Liu [1,2], Lirong Liu [1] and Guowei Liu [1]

1 School of Environmental Science and Engineering, Guangzhou University, Guangzhou 510006, China; huangxuexia66@163.com (X.H.); tfxiao@gzhu.edu.cn (T.X.); wlz2016@gzhu.edu.cn (L.W.); liuyu@gzhu.edu.cn (Y.L.); leonliutiming@e.gzhu.edu.cn (L.L.); liuguowei@e.gzhu.edu.cn (G.L.)
2 Linköping University—Guangzhou University Research Center on Urban Sustainable Development, Guangzhou University, Guangzhou 510006, China
3 Guangdong Provincial Key Laboratory of Radionuclides Pollution Control and Resources, Guangzhou University, Guangzhou 510006, China
4 Key Laboratory for Water Quality and Conservation of the Pearl River Delta, Ministry of Education, Guangzhou University, Guangzhou 510006, China
5 Environmental Engineering Program, Department of Civil Engineering, Auburn University, Auburn, Alabama 36849, USA; zhaodon@auburn.edu
6 Guangxi Zhuang Autonomous Region Environmental Monitoring Station, Nanning 530028, China; lining1972@sohu.com
7 Ecological Environment Information System and Big Data Research Team, Guangdong University of Technology, Guangzhou 510006, China; liujycust@163.com
* Correspondence: ldggq@gzhu.edu.cn

Table S1. Longitudinal and latitudinal positions of the sampling sites.

Sites No.	Longitude	Latitude	Sites No.	Longitude	Latitude
S1	111°21'14'' E	23°28'27'' N	S2	111°25'07''E	23°28'06'' N
S3	111°29'50''E	23°25'30''N	S4	111°31'00''E	23°21'07''N
S5	111°33'22''E	23°17'33''N	S6	111°34'26''E	23°15'25''N
S7	111°32'02''E	23°13'31''N	S8	111°34'12''E	23°11'25''N
S9	111°38'52''E	23°09'56''N	S10	111°44'37''E	23°08'33''N
S11	111°49'37''E	23°08'00''N	S12	111°52'06''E,	23°08'36''N
S13	111°55'18''E	23°07'29''N	S14	111°58'47''E	23°06'08''N
S15	112°04'07''E	23°04'37''N	S16	112°07'33''E	23°05'01''N
S17	112°12'14''E	23°04'43''N	S18	112°16'33''E	23°07'36''N
S19	112°20'27''E	23°09'55''N	S20	112°23'33''E	23°07'55''N
S21	112°23'55''E	23°04'08''N	S22	112°31'55''E	23°04'45''N
S23	112°34'46''E	23°08'15''N	S24	112°37'14''E	23°10'58''N
S25	112°43'22''E	23°10'05''N	S26	112°47'24''E	23°08'53''N
S27	112°48'02''E	23°05'35''N	S28	112°49'09''E	23°02'30''N
S29	112°50'60''E	22°59'25''N	S30	112°54'19''E	22°55'27''N
S31	112°55'23''E	22°51'17''N	S32	113° 00'25''E	22°48'20''N
S33	113°04'18''E	22°47'16''N	S34	113°05'31''E	22°42'28''N

S35	113°06'09"E	22°39'17"N	S36	113°08'58" E	22°36'18" N
S37	113°10'46"E	22°32'29"N	S38	113°10'36"E	22°29'21"N
S39	113°15'03"E	22°26'59"N	S40	113°14'57"E	22°24'02"N
S41	113°17'58"E	22°21'09"N	S42	113°20'20"E	22°16'20"N
S43	113°23'12"E	22°12'45"N			

Table S2. Metals/metalloids concentrations in water from open publications (μg L^{-1}).

Location	V	Co	Cr	Ni	Cu	Mn	Zn	Cd	Pb	As	Sb	Tl	References
Xijiang River,China	0.60	0.48	2.37	1.73	3.08	20.9	18.34	0.17	1.03	1.72	0.65	0.03	This study
Beijiang River,China	0.27	- [a]	11.01	1.00	3.02	9	18.13	0.12	1.69	6.48	1.27	0.01	(Li et al., 2018b; Song et al., 2011; Zhou et al., 2017)
Jinjiang River,China	0.97	0.12	0.53	1.70	1.45	11.1	1.98	0.02	0.39	4.84	0.14	0.00	(Liu et al., 2018)
Xiangjiang River,China	-	-	0.12	0.55	0.87	20.29	0.0	0.15	0.0	4.84	0.0	0.03	(Li et al., 2018a)
Yangtze River,China	10.5	-	20.9	13.4	10.7	5.4	9.4	4.7	55.1	13.2	65.3	-	(Wu et al., 2009)

Note: a: Not detected

Table S3. Correlation analysis among different metals/metalloids in the water samples (n = 43).

metals	V	Co	Cr	Ni	Cu	Mn	Zn	Cd	Pb	As	Sb	Tl
V	1											
Co	0.512**	1										
Cr	0.282	0.070	1									
Ni	0.339*	0.220	0.817**	1								
Cu	0.290	0.108	0.871**	0.855**	1							
Mn	0.201	0.033	0.826**	0.854**	0.880**	1						
Zn	0.168	-0.041	0.809**	0.870**	0.871**	0.911**	1					
Cd	0.201	0.049	0.870**	0.845**	0.809**	0.820**	0.863**	1				
Pb	0.181	0.066	0.862**	0.794**	0.833**	0.827**	0.849**	0.879**	1			
As	0.296	0.059	0.950**	0.848**	0.846**	0.819**	0.826**	0.864**	0.833**	1		
Sb	0.385**	0.168	0.363*	0.234	0.097	0.161	0.092	0.235	0.293	0.389*	1	

Tl	0.036	-0.118	0.613**	0.426**	0.376*	0.517**	0.440**	0.583**	0.530**	0.567**	0.446**	1

* Correlation is significant at $P < 0.05$. ** Correlation is significant at $P < 0.01$.

Table S4. Metals/metalloids concentrations in sediments from open publications (mg kg^{-1} d. w.).

Location	V	Co	Cr	Ni	Cu	Mn	Zn	Cd	Pb	As	Sb	Tl	References
Xijiang River,China	59.8	14.14	88.43	45.24	70.43	689.5	466.0	5.09	87.82	83.30	7.54	1.21	This study
Beijiang River	60.3	9.6	75.2	31.7	89.0	- [a]	383	6.3	225	83.59	39.0	1.70	(Gao et al., 2012; Gao et al., 2008; Li et al., 2018b; Li et al., 2019)
Yangtze River,China	-	-	87.8	40.9	51.6	-	140	1.53	45.2	15.9	-	-	(Yang et al., 2009)
Jinjiang River	93.8	11.6	58.66	24.47	26.04	787	104	0.51	23.81	6.20	0.55	0.44	(Liu et al., 2018)
Hejiang River,China	65.4	9.58	44.3	21.7	43.5	975.8	187.5	1.42	78.48	83.1	13.5	0.97	(Ning et al., 2017)
Background values of the sediments of the Pearl River, China	105	18	86	35	38	820	85	0.09	30	17	1.35	0.52 [b]	(Chi and Yan, 2007)

Note: a: Not detected; b: the background value of soil in Guangdong Province.

Table S5. Correlation analysis among different metals/metalloids in the sediment samples (n = 43).

metals	V	Co	Cr	Ni	Cu	Mn	Zn	Cd	Pb	As	Sb	Tl
V	1											
Co	0.824**	1										
Cr	0.232	0.360*	1									
Ni	0.229	0.320*	0.784**	1								
Cu	0.190	0.385*	0.815**	0.904**	1							
Mn	0.283	0.314*	0.873**	0.780**	0.789**	1						
Zn	0.208	0.219	0.654**	0.713**	0.693**	0.750**	1					
Cd	0.036	0.250	0.889**	0.788**	0.844**	0.851**	0.698**	1				
Pb	0.226	0.407*	0.744**	0.652**	0.761**	0.774**	0.633**	0.799**	1			

As	0.185	0.263	0.893**	0.786**	0.751**	0.807**	0.677**	0.776**	0.602**	1		
Sb	0.203	0.187	0.318*	0.497**	0.381*	0.385*	0.528**	0.291	0.107	0.407**	1	
Tl	-0.108	-0.045	0.484**	0.440**	0.512**	0.508**	0.653**	0.594**	0.526**	0.412**	0.401**	1

* Correlation is significant at $P < 0.05$

** Correlation is significant at $P < 0.01$

Table S6. Correlation analysis among different metals/metalloids in the *Corbicula fluminea* samples (n = 34).

metals	V	Co	Cr	Ni	Cu	Mn	Zn	Cd	Pb	As	Sb	Tl
V	1											
Co	0.329	1										
Cr	-0.060	0.066	1									
Ni	-0.126	-0.087	0.134	1								
Cu	0.297	0.437**	0.256	-0.159	1							
Mn	0.319	**0.581****	0.183	0.195	0.499**	1						
Zn	0.317	0.527**	0.028	0.079	**0.574****	0.556**	1					
Cd	0.414*	0.428*	0.316	-0.020	**0.678****	0.575**	0.651**	1				
Pb	0.244	0.490**	0.177	0.078	0.519**	0.430*	**0.588****	0.480**	1			
As	0.489**	0.650**	0.258	0.029	0.617**	0.664**	0.630**	**0.713****	0.609**	1		
Sb	0.216	0.137	0.002	-0.115	0.272	0.266	0.272	0.530**	0.218	0.466**	1	
Tl	0.183	0.468**	-0.016	0.292	0.247	0.288	0.536**	0.436*	0.519**	0.528**	0.241	1

* Correlation is significant at $P < 0.05$

** Correlation is significant at $P < 0.01$

Table S7. Correlations between *Corbicula fluminea* soft tissue and single metal/metalloid in water and sediment (n = 34).

		V	Co	Cr	Ni	Cu	Mn	Zn	Cd	Pb	As	Sb	Tl
Water													
Sediment	r	0.580**	.566**	0.613**	0.830**	0.714**	0.754**	0.710**	0.789**	0.650**	0.726**	0.315	0.674**

	P	0.000	.000	0.000	0.000	0.000	0.000	0.000	0.000	0.000	0.000	0.070	0.000
Water													
Soft tissue	r	0.021	0.012	0.131	0.253	0.399*	0.389*	0.425*	0.344*	0.364*	0.373*	0.126	0.195
	P	0.907	0.948	0.460	0.149	0.020	0.023	0.012	0.047	0.034	0.030	0.477	0.269
Sediment													
Soft tissue	r	0.326	0.378*	0.341*	0.208	0.557**	0.484**	0.532**	0.543**	0.615**	0.599**	0.154	0.387*
	P	0.060	0.027	0.048	0.239	0.001	0.004	0.001	0.001	0.000	0.000	0.383	0.024

* Correlation is significant at $P < 0.05$. ** Correlation is significant at $P < 0.01$.

Table S8. Geo-accumulation index, potential ecological risk, and average bioconcentration factors (*BAF*) and biota-sediment accumulation factors (*BSF*) for metals/metalloids in *Corbicula fluminea* soft tissue from the Xijiang River.

	V	Co	Cr	Ni	Cu	Mn	Zn	Cd	Pb	As	Sb	Tl
The soil background value in China	82	13	65	26	24	600	68	0.09	23	10	0.8	0.6
BAF	2583	3319	446	703	20503	2949	14889	23706	2354	2684	106	3667
BSF	0.0259	0.1106	0.0121	0.0264	0.8505	0.0862	0.5609	0.8224	0.0269	0.0589	0.0093	0.0894
I_{geo}	-1.3	-1.8	-0.3	-1.0	0.2	-0.9	1.7	5.2	1.8	4.7	5.0	-0.4
E_r^i	1.5	5.4	2.7	8.7	14.7	1.1	6.9	1695.1	19.1	83.3	65.9	20.2

Table S9. Component Score Coefficient Matrix.

	Principal component				Principal component		
	PC1	PC2	PC3		PC1	PC2	PC3
V	-.091	.506	.072	Zn	.030	-.031	.278
Co	-.015	.476	-.065	Cd	.206	-.093	-.130
Cr	.181	.012	-.120	Pb	.225	.019	-.282
Ni	.094	.024	.101	As	.127	-.006	.016
Cu	.149	.011	-.034	Sb	-.217	.060	.753
Mn	.147	.017	-.028	Tl	.043	-.217	.264

Table S10. Scores of principal factors on samples.

Sites	Fac1	Fac2	Fac3	Sites	Fac1	Fac2	Fac3
s1	0.2822	0.36077	0.43796	s23	-0.78488	-0.64729	-0.46235
s2	0.2576	0.73753	0.54002	s24	-1.33769	-0.72188	-1.12638
s3	-0.1096	0.31235	1.76552	s25	-0.6648	-0.61693	-0.41571
s4	-0.42263	0.35405	1.16129	s26	0.37433	-0.60954	0.93466
s5	-0.15686	1.44214	-0.89864	s27	0.17713	-0.79131	2.62668
s6	-0.94413	1.13447	0.38007	s28	0.33789	1.06578	-1.35354
s7	-0.80983	2.02099	0.37011	s29	0.27664	0.90999	-1.74097
s8	-0.99618	1.82836	0.20125	s30	-0.03633	0.48077	-0.77595
s9	-0.98855	0.17027	0.02497	s31	3.15661	-0.05219	-1.18016
s10	-1.0306	-0.06877	0.1236	s32	1.76062	0.12964	1.10587
s11	-0.46292	-0.01729	-0.92202	s33	1.81033	-0.22798	0.05528
s12	-1.12063	-0.32682	0.09538	s34	0.99271	-0.47299	0.46092
s13	-1.28808	-0.78915	-0.89697	s35	0.21289	-0.55175	0.12131
s14	-0.50419	-0.86835	-1.22221	s36	2.08919	0.26609	0.12154
s15	0.63513	-1.54047	1.2323	s37	1.13805	0.02427	0.19563
s16	0.54357	-1.90023	1.59145	s38	0.44163	-0.27698	-0.26227
s17	0.37441	-1.91011	-0.86749	s39	-0.03334	-0.22031	-0.4469
s18	-0.7017	-0.59299	0.25762	s40	0.03447	-0.30315	-0.94418
s19	-0.79613	-0.42281	-0.02847	s41	0.39322	-0.40633	-1.14043
s20	-1.06	-0.49452	-0.13007	s42	1.38536	2.6628	-0.91706
s21	-1.03818	-0.64448	-0.3618	s43	-0.82392	2.21404	2.56154
s22	-0.56282	-0.6397	-0.27139				

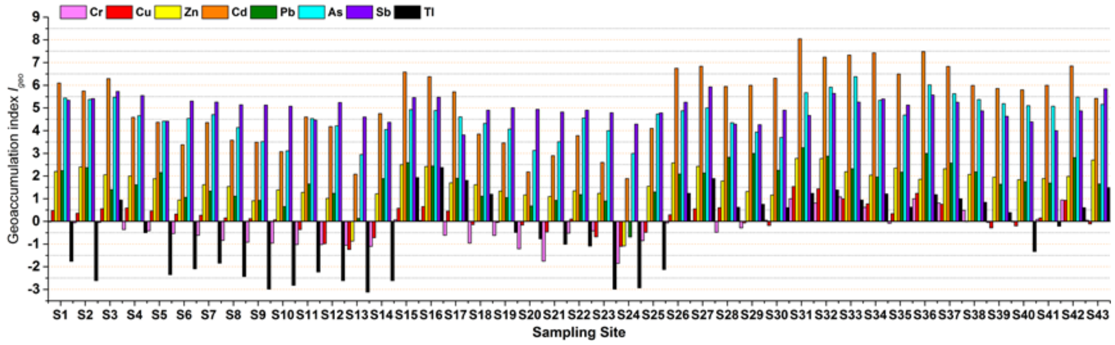

Figure S1. Box plots of geo-accumulation index of 8 metals/metalloids in the sediments from Xijiang River.

Chi, Q., Yan, M., 2007. Handbook of Elemental Abundance for Applied Geochemistry. Geological Publishing House, Beijing China (In Chinese).

Gao, B., et al., 2012. The distribution, accumulation and potential source of seldom monitored trace elements in sediments of Beijiang River, South China. Water Science and Technology. 65, 2118-2124.

Gao, B., et al., 2008. Ecological risk assessment of thallium pollution in the surface sediment of Beijiang River. Ecology and Environmental Sciences. 17, 528-532.(In Chinese).

Li, D., et al., 2018a. Evaluating a 5-year metal contamination remediation and the biomonitoring potential of a freshwater gastropod along the Xiangjiang River, China. Environmental Science and Pollution Research. 25, 21127-21137.

Li, R., et al., 2018b. The distribution and partitioning of trace metals (Pb, Cd, Cu, and Zn) and metalloid (As) in the Beijiang River. Environmental Monitoring and Assessment. 190.

Li, R., et al., 2019. Reconstructing the historical pollution levels and ecological risks over the past sixty years in sediments of the Beijiang River, South China. Science of the Total Environment. 649, 448-460.

Liu, X., et al., 2018. Distribution and risk assessment of metals in water, sediments, and wild fish from Jinjiang River in Chengdu, China. Chemosphere. 196, 45-52.

Ning, Z., et al., 2017. Spatial distribution characteristics, sources and potential ecological risk of heavy metals in sediments of the Hejiang River. China Environmental Science. 37, 3036-3047(In Chinese).

Song, M. W., et al., 2011. Water quality of a tributary of the Pearl River, the Beijiang, Southern China: implications from multivariate statistical analyses. Environmental Monitoring and Assessment. 172, 589-603.

Wu, B., et al., 2009. Preliminary Risk Assessment of Trace Metal Pollution in Surface Water from Yangtze River in Nanjing Section, China. Bulletin of Environmental Contamination and Toxicology. 82, 405-409.

Yang, Z., et al., 2009. Distribution and speciation of heavy metals in sediments from the mainstream, tributaries, and lakes of the Yangtze River catchment of Wuhan, China. Journal of Hazardous Materials. 166, 1186-1194.

Zhou, K., et al., 2017. Investigation and Analysis on Water Quality of Beijiang River in 2016. City and Town Water Supply. 22-25(In Chinese).

© 2019 by the authors. Submitted for possible open access publication under the terms and conditions of the Creative Commons Attribution (CC BY) license (http://creativecommons.org/licenses/by/4.0/).
